# Metacognitive monitoring and control in visual change detection: Implications for situation awareness and cognitive control

**DOI:** 10.1371/journal.pone.0176032

**Published:** 2017-09-15

**Authors:** Ken I. McAnally, Adam P. Morris, Christopher Best

**Affiliations:** 1 Aerospace Division, Defence Science and Technology Group, Fishermens Bend, Victoria, Australia; 2 School of Psychological Sciences, University of Melbourne, Parkville, Victoria, Australia; 3 Neuroscience Program, Biomedicine Discovery Institute, Department of Physiology, Monash University, Clayton, Victoria, Australia; Universidade Federal do Rio de Janeiro, BRAZIL

## Abstract

Metacognitive monitoring and control of situation awareness (SA) are important for a range of safety-critical roles (e.g., air traffic control, military command and control). We examined the factors affecting these processes using a visual change detection task that included representative tactical displays. SA was assessed by asking novice observers to detect changes to a tactical display. Metacognitive monitoring was assessed by asking observers to estimate the probability that they would correctly detect a change, either after study of the display and before the change (judgement of learning; JOL) or after the change and detection response (judgement of performance; JOP). In Experiment 1, observers failed to detect some changes to the display, indicating imperfect SA, but JOPs were reasonably well calibrated to objective performance. Experiment 2 examined JOLs and JOPs in two task contexts: with study-time limits imposed by the task or with self-pacing to meet specified performance targets. JOPs were well calibrated in both conditions as were JOLs for high performance targets. In summary, observers had limited SA, but good insight about their performance and learning for high performance targets and allocated study time appropriately.

## Introduction

Metacognition is the knowledge about and regulation of one’s own cognitive processes [1]. In many work environments, metacognition is important for planning, managing attention, making decisions and monitoring performance [2–4]. The prediction of human performance in complex work environments–particularly those characterised by multi-tasking–requires a better understanding of the manner in which cognitive performance is monitored and attentional resources are controlled by metacognitive processes.

The interaction between the monitoring and control functions of metacognition may be demonstrated in the context of studying for an examination (see [4] for a review). While studying, learners make judgements of learning (JOLs) by assessing their current state of knowledge (monitoring; [5]). These judgements are used to terminate study (control) when the material is thought to have been sufficiently learned. Conversely, the effort expended in committing information to memory (control) affects judgements of the degree to which that information has been learned (monitoring). For example, according to the "effort heuristic", items which require more effort to study are judged by learners to be less well learned [6].

Similar relationships between metacognitive monitoring and control may operate in the conduct of many kinds of safety-critical work. For example, air traffic controllers build their understanding of the positions and movements of aircraft (i.e., their situation awareness; SA) based on information presented on their displays. Situation awareness has been defined as "the perception of the elements in the environment within a volume of time and space, the comprehension of their meaning, and the projection of their status in the near future" [7]. When their understanding of the flow of traffic in one sector of airspace is judged to be sufficient, they may redirect their attention to another sector or to a different task altogether (e.g., communicating with their supervisor). Crucially, any mismatch between their objective and perceived knowledge is likely to lead to the inappropriate control of attention and in turn, to an increase in the likelihood of human error. Indeed, a comparison [8] between a subjective measure of SA (the Situational Awareness Rating Technique [9]) and an objective measure (the Situation Awareness Global Assessment Technique [10]) has shown that while the subjective measure was correlated with confidence and perceived performance, it was not correlated with the objective measure. This suggests that operators are often not aware of gaps in their knowledge.

The magnitude of metacognitive (mis)calibration could depend on the parameters of the operational context such as the likelihood that important information will change, time pressure during the acquisition of knowledge, whether study is self-paced or under the control of task factors, and acceptable tolerance for error (e.g., zero-tolerance for errors in air traffic control, compared with a more relaxed tolerance in a manufacturing line). In addition, the requirements to self-monitor knowledge acquisition and to regulate study behaviour might themselves affect SA or reduce efficiency due to a division of finite cognitive resources.

The current study addresses these issues in the context of a change-detection task involving stimuli that are representative of the radar displays found in air-traffic control and similar work environments. Previous studies in cognitive psychology have found observers to be surprisingly poor in detecting large changes to visual scenes if transients associated with those changes are masked (see [11,12] for reviews). This "change blindness" has been interpreted as evidence of either relatively sparse encoding of the visual scene [13], or difficulty in retaining and recalling [14] or comparing [15] multiple items in visual short-term memory (VSTM).

A corollary of the surprisingly poor detection of changes is the belief that changes would be easy to detect. This belief leads to overconfidence in one's ability to detect changes, a phenomenon referred to as "change blindness blindness" [16–18]. This overconfidence may be based on the phenomenological experience of an extensive visual awareness [19] or on observers' beliefs about their ability to understand meaning and structure in scenes [20].

Change detection tasks have been employed in a study of work environments, where the extent to which operators are able to detect changes to task-relevant visual information presented on their displays has been interpreted to reflect the extent to which they had awareness of the information presented and its meaning, i.e., SA [21]. In tactical and aeronautical contexts, a lack of SA could lead to risk of personal harm or other adverse outcomes such as the collision of aircraft. Further, over-confidence on the part of operators regarding their ability to detect important changes (i.e., change blindness blindness) may result in failure to allocate sufficient attention to displays to achieve desired task objectives. Accordingly, it is crucial to understand the fundamental cognitive factors that operate in change-detection tasks that are of comparable complexity and appearance to their real-world counterparts.

The aim of the current study was to measure objective and subjective performance in radar-like change detection tasks and to examine factors that might modulate metacognitive accuracy and performance. These factors included the likelihood of a change occurring (Experiment 1) and the degree of time pressure during acquisition of knowledge (Experiment 2). In addition, we examined costs associated with the need to self-monitor the acquisition of knowledge on both objective performance and metacognitive accuracy (Experiment 2). To ensure that our results are of relevance to a broad range of applied domains, participants in our experiments were non-experts rather than trained operators. Future work will be needed to establish how the findings presented below may be modulated during the acquisition of domain-specific expertise.

## Experiment 1

### Method

#### Participants

Ten non-expert observers (6 male, 4 female) participated. All were staff or vacation students at the Defence Science and Technology Organisation (DSTO). Their average age was 22.1 years (s.d. = 1.4 years). All had normal or corrected-to-normal vision. All gave written informed consent before participating and were allowed to withdraw from the study at any time. The project was approved by the Chief of Air Operations Division as a delegate of the Australian Defence Human Research Ethics Committee, in accordance with the Australian National Statement on Ethical Conduct in Human Research [22].

#### Change detection task

**Fig 1:**
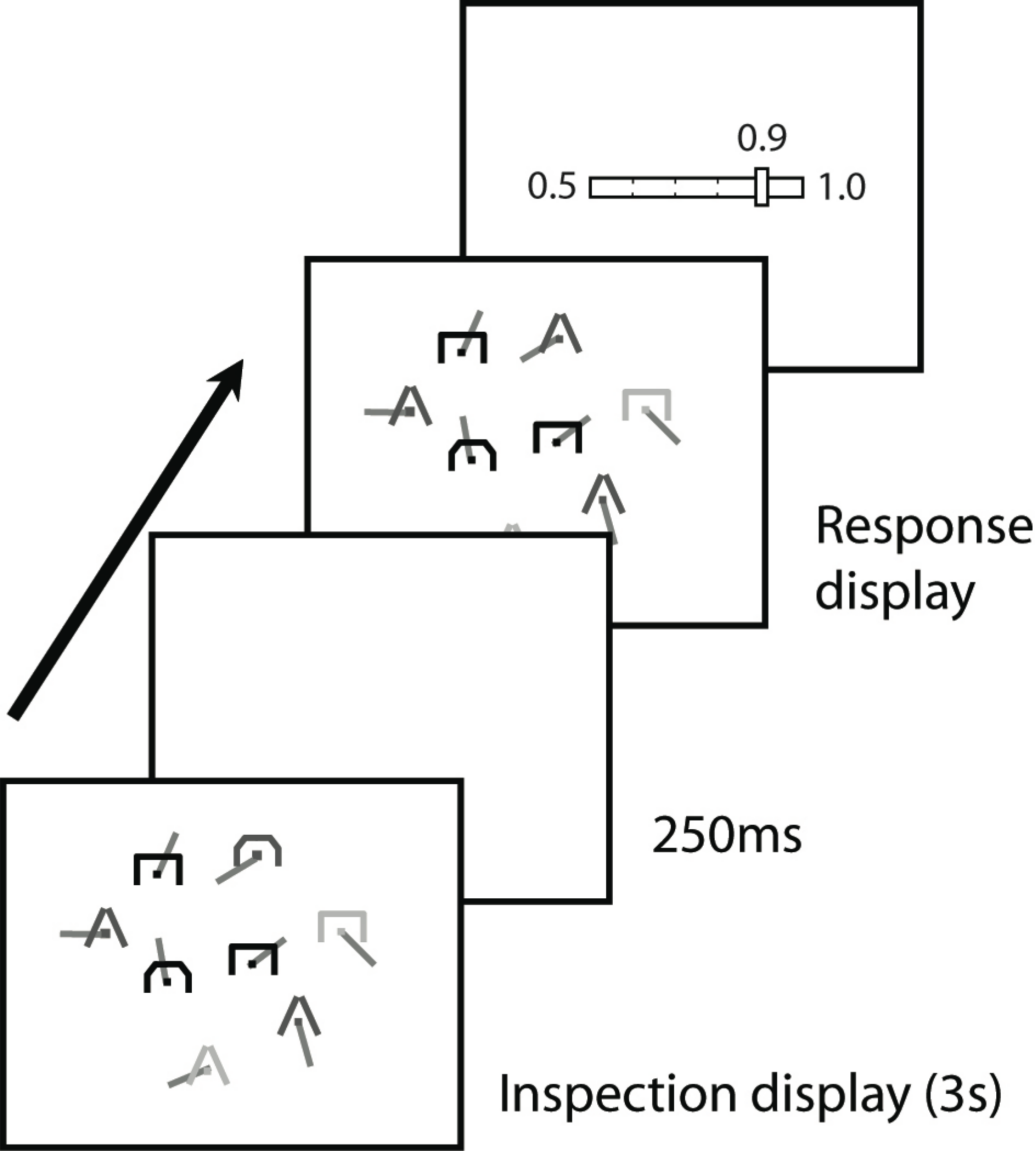
Schematic of the trial sequence. Eight symbols were shown in each trial. On each trial, the shape of one of the symbols may change during the mask period. Observers were required to detect and localise the change and to make a judgement of their performance (JOP) in the detection task by moving the slider to their perceived probability of a correct response.

The displays used in the change detection task were modelled on tactical displays used in aeronautical and military environments (Fig 1). Each display consisted of a set of eight symbolic entities based on the Hostile, Ambiguous, Friendly, Unknown (HAFU) symbology [23], in which the entity identity and movement direction are represented by different symbol features (colour and shape, and the orientation of a leader line). The participants, however, were not instructed about the semantic content of the symbology.

Stimuli were generated using OpenGL and displayed on a HP2465 24" LCD monitor. Viewing distance was approximately 60 cm. Each trial began with a 3-second inspection interval during which observers viewed a static display containing eight symbols. Each symbol was positioned at a random position within a central region of the display subtending 30° × 30°. Individual symbols subtended approximately 0.6° × 0.6° and were defined by their colour, shape, and the orientation of a leader line of length 1.3° emanating from its centre. The colour of each symbol was chosen at random, with replacement, from a set of three (blue, yellow, red). The shape of each symbol was chosen at random, with replacement, from a set of three (open triangle, open rectangle, open semi-circle). Line orientation was selected at random.

Immediately following the initial display, a uniform grey mask was presented for 0.25 seconds. Immediately following the mask, a second display was presented which was either identical to the initial display, or identical with the exception that the shape of one of the symbols was changed to another from the set of possible shapes. Two conditions of change probability (25%, 75%) were presented in separate blocks of trials. Crucially, in order to avoid the provision of implicit feedback and potential for probability matching, observers were naive with respect to the probabilities of change in these conditions. Observers completed six blocks of 40 trials in each condition of change probability. The order of presentation of blocks was counterbalanced within and across observers.

The observers' task was to indicate with a mouse click whether or not one of the symbols had changed in shape between the first and second displays (a yes/no task). Observers then indicated their confidence in their detection response (i.e., a judgement of performance; JOP) on a continuous scale from 0.5 (chance) to 1.0 (certain). Observers were then required to indicate which of the eight symbols had changed by selecting it with the mouse. This identification response was required for all trials, including those where no change was detected, given the possibility that a change may have been missed. Observers were given an unlimited amount of time to respond and received no feedback with regard to their performance.

#### Assessment of subjective judgements

The accuracy of subjective judgements was analysed using a decomposition of Brier scores [24] into components of calibration and resolution [25,26]. Calibration reflects the degree to which subjective and objective probabilities agree across the range, while resolution reflects the degree to which observers are able to discriminate between events where objective probabilities differ [26,27]. JOPs for each observer were sorted into bins (of width 0.1) of increasing confidence from .5 (chance) to 1.0 (perfect performance). The subjective confidence for each bin is defined as the mid-point of the bin. The mean objective performance for trials in each bin was calculated and plotted against the JOP associated with the bin as calibration curves.

**Fig 2:**
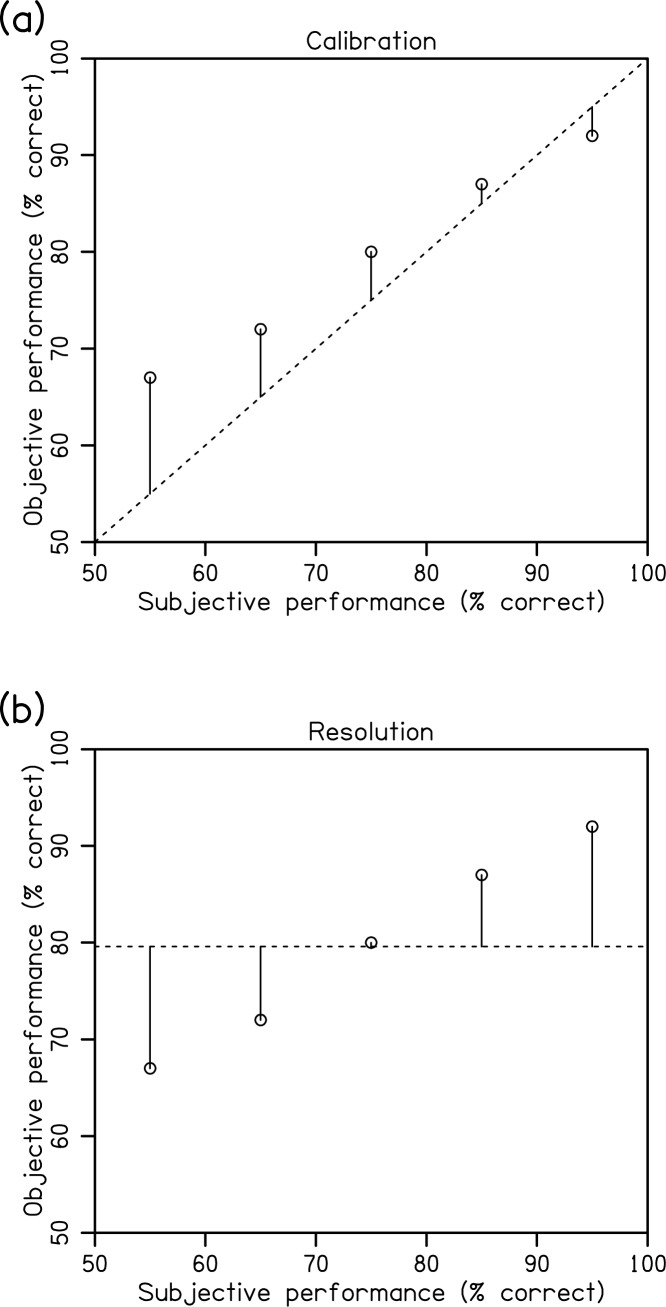
Schematic of calibration and resolution metrics. (a) Calibration is the weighted sum of squared deviations between the calibration curve and the positive diagonal. (b) Resolution is the weighted sum of squared deviations between the calibration curve and the average objective performance. Hypothetical mean objective data are shown.

Calibration is a weighted sum of squared deviations between the calibration curve and the positive diagonal, and reflects the error of subjective judgements (Fig 2).

$$\mathrm{Calibration}=\frac{1}{N}\sum_{k=1}^{K}n_{k}{\left(s_{k}-{\bar{o}}_{k}\right)}^{2}$$

where *N* is the total number of trials, *K* = number of bins of subjective judgement, *n*_*k*_ = number of trials in bin *k*, *s*_*k*_ = subjective judgement associated with bin *k*, and ${\bar{o}}_{k}$ = average objective performance of trials in bin *k*. Calibration varies from zero for perfect agreement, to a maximum of .20 in Experiment 1 and .47 in Experiment 2.

Resolution is a weighted sum of squared deviations between the calibration curve and the average objective performance, and reflects the degree to which observers can discriminate between trials of low and high objective performance (see Fig 2).

$$\mathrm{Resolution}=\frac{1}{N}\sum_{k=1}^{K}n_{k}{\left({\bar{o}}_{k}-\bar{o}\right)}^{2}$$

where $\bar{o}$ = average objective performance.

The resolution score varies from zero to a maximum equal to the uncertainty component of the Brier score [26].

$$\mathrm{Uncertainty}=\bar{o}\left(1-\bar{o}\right)$$

We divided resolution by uncertainty to give normalised resolution, which is the between-category portion of the overall variance, comparable to *η*^*2*^ [26].

We also computed the average confidence error, which is also called overconfidence [28] or calibration-in-the-large [29].

$$\mathrm{Average}\;\mathrm{confidence}\;\mathrm{error}=\bar{s}-\bar{o}$$

where $\bar{s}$ = average subjective judgement.

The ability of observers to discriminate between correct and incorrect detections is sometimes analysed using type-2 signal detection theory (SDT). In this framework, 'hit trials' are those in which the observer expressed high confidence in their detection response and was indeed correct. The remaining high-confidence trials–that is, those in which their detection response was actually incorrect–are considered ‘false-alarms’ [30]. A complication with this approach, however, is that type-2 sensitivity (*d')* for a given condition depends on the associated objective (i.e. type 1) sensitivity, and on types-1 and -2 response biases [31]. This dependence hinders the interpretation of type-2 *d'* as a measure of metacognitive sensitivity. A recent study [32] has described a method for examining metacognitive sensitivity in 2-alternate forced-choice procedures using *meta d'*, i.e., the type-1 *d'* which would give rise to the observed type-2 receiver operating characteristic if there was no loss of information between type-1 and -2 responses. We calculated type-1 *d'* assuming unequal variances and meta *d'* using the software described in [32].

#### Statistical analysis

Means were analysed using paired *t*-tests or repeated-measures analyses of variance (ANOVAs). An alpha level of .05 was used for all statistical tests.

### Results

#### Objective change detection performance

Trials from each condition of change probability were partitioned into change and no-change trials. Change detection accuracies (correct detections and rejections) for each condition of change probability are shown in Table 1. Accuracy (correct detections and rejections) was analysed with a 2-way repeated-measures ANOVA with variables of Change (change, no change) and Change Probability (25, 75%). There was no significant main effect of Change, *F*(1,9) = 2.60, *p* = .14, *η*_*p*_^*2*^ = .22, or Change Probability, *F*(1,9) = 0.004, *p* = .95, *η*_*p*_^*2*^ < .001, and no significant interaction between these variables, *F*(1,9) = 0.02, *p* = .89, *η*_*p*_^*2*^ = .002. Consistent with the absence of a main effect of Change Probability on accuracy, type-1 *d'* was not significantly different between these conditions (Table 1). Following the detection response and confidence judgement, observers were required to identify the symbol that had changed. Observers did not identify all detected changes correctly. However, the rate at which detected changes were correctly identified did not differ between conditions of Change Probability (Table 1).

**Table 1:** Accuracy of change detection and identification.

	25% changes	75% changes	*t*(9)	*p*
**Correct detections (%)**	76.0 (3.9)	75.7 (4.2)	-	-
**Correct rejections (%)**	82.2 (2.2)	82.3 (3.4)	-	-
**Type-1 *d'***	1.90 (0.27)	1.91 (0.27)	0.11	.91
**Detections correctly identified (%)**	83.7 (3.8)	86.6 (3.4)	1.24	.25

Note: Standard errors of the means are shown in parentheses.

#### Metacognitive monitoring—Judgements of performance

**Fig 3:**
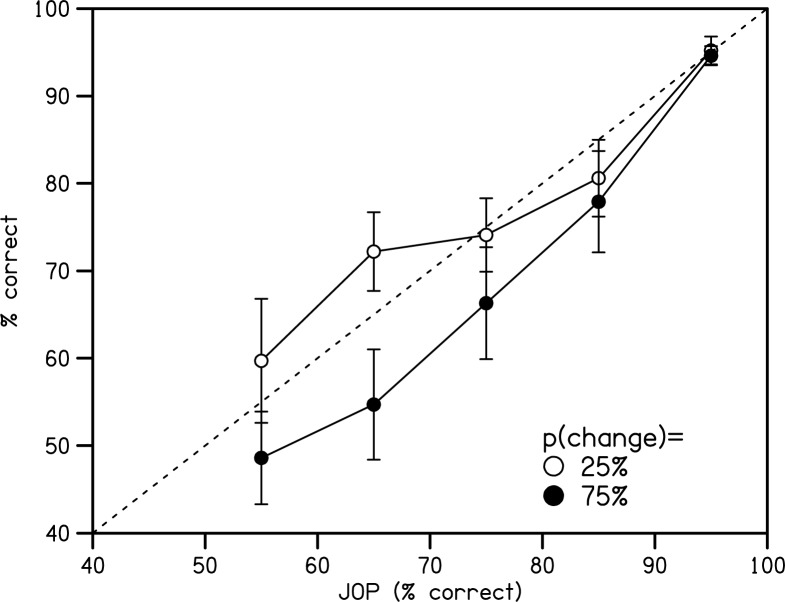
Calibration curves. Calibration curves for the two conditions of change probability in Experiment 1. Errors bars indicate standard errors of the means.

JOPs were elicited by asking observers to rate their confidence in their detection decisions on a scale from .5 (chance) to 1.0 (certain). Inspection of Fig 3 shows that these judgements were reasonably well calibrated with objective performance (i.e., close to the positive diagonal).

Metrics of metacognition in change detection are shown in Table 2. Average JOPs were analysed in a 2-way repeated-measures ANOVA with the independent variables of Change (change, no change) and Change Probability (25, 75%). The main effect of Change was significant, *F*(1,9) = 27.6, *p* = .001, *η*_*p*_^*2*^ = .75, but the main effect of Change probability and its interaction with Change were not significant, *F*(1,9) = 0.38, *p* = .55, *η*_*p*_^*2*^ = .04, and *F*(1,9) = 0.36, *p* = .56, *η*_*p*_^*2*^ = .04, respectively.

**Table 2:** Metrics of metacognition in change detection.

	25% changes	75% changes	*t*(9)	*p*
**JOP (change)** **JOP (no change)** **Av. confidence error (change)**	.828 (.023) .783 (.017) .068 (.042)	.827 (.021) .775 (.018) .070 (.047)	- - -	- - -
**Av. confidence error (no change)**	-.040 (.034)	-.048 (.043)	-	-
**Calibration**	.015 (.004)	.024 (.007)	1.67	.13
**Normalised resolution**	.104 (.010)	.188 (.016)	5.65	< .001
**Meta *d'***	1.48 (0.16)	1.55 (0.14)	0.39	.70
**Type-1 *d'—*meta *d'***	0.42 (0.26)	0.36 (0.31)	0.30	.77

Note: Standard errors of the means are shown in parentheses.

Average confidence error was analysed in a 2-way repeated-measures ANOVA with the independent variables of Change (change, no change) and Change Probability (25, 75%). There was a significant main effect of Change, *F*(1,9) = 8.17, *p* = .02, *η*_*p*_^*2*^ = .48, with trends for overconfidence in the change trials and for underconfidence in the no-change trials. The main effect of Change Probability and its interaction with Change were not significant, *F*(1,9) = 0.05, *p* = .82, *η*_*p*_^*2*^ = .006, and *F*(1,9) = 0.14, *p* = .72, *η*_*p*_^*2*^ = .01, respectively. For each condition of Change and Change Probability, the average confidence error was not significantly different from zero, *t*(9) ≤ 1.61, *p* ≥ .14.

Calibration and resolution scores were generated for each observer from all trials in each condition of Change Probability. Calibration was not significantly different between these conditions (Table 2). Normalised resolution was significantly higher in the condition with more frequent changes, indicating a better ability to differentiate trials of high and low objective performance (Table 2).

Average *meta d'* was not significantly different between conditions of Change Probability (Table 2). On average, *meta d'* was around 0.4 lower than type-1 *d'*, indicating a small departure from optimal metacognitive sensitivity, given the limitations imposed by type-1 (change detection) sensitivity. This difference was not significantly different between conditions of Change Probability (Table 2).

### Discussion

Average change detection rates were around 75%, indicating imperfect SA of the information presented on the display. Average change detection and identification rates were similar for 25% and 75% base rates of change.

Observers' JOPs were generally well calibrated to their objective performance. There were two significant effects observed for metacognition. First, observers reported significantly higher confidence in the correctness of their responses (JOPs) on change trials than on no-change trials, even though their relative objective performance in these two conditions was (non-significantly) the other way around (Table 1; compare correct detections and rejections). This suggests that the subjective experience of evidence of a change is more compelling than the absence of evidence on no-change trials, reflecting at least partial insight into the phenomenon of change blindness.

Second, the ability of observers to resolve poor from good performance, as measured by resolution, was significantly better when changes were more frequent. This is also consistent with the possibility that the subjective experience of a change provides a stronger meta-cognitive signal than the absence of such an experience on no-change trials. In contrast, *meta d'*–which is conceptually similar to resolution–showed no significant effect of change probability. The reason for this difference across metrics is not clear but could be related to the different assumptions in the two measures; for example, meta *d'* assumes that evidence is noisy (Gaussian) and that detection (type 1) and metacognition (type 2) decisions are based on the same evidence dimension, whereas resolution makes no assumptions about representation or decision processes.

The absence of significant overconfidence in the results of Experiment 1 is not consistent with the gross overconfidence reported in studies of change blindness blindness, e.g., [16]. It is possible that the previously reported overconfidence in the ability to detect changes is present only for natural scenes where observers have a phenomenological experience of an extensive visual awareness [19] or where scenes contain information which is meaningful to the observer [20]. With regard to this latter possibility, the stimuli of the present study were symbolic and abstract in nature, so the encoding of shape may have been effortful. According to the effort heuristic [6], increased effort during study is associated with decreased judgements of learning. It is possible that the effort applied in encoding symbol shapes resulted in a lowering of confidence to a level where it matched objective performance well. Experienced operators (e.g., military personnel) who are familiar with these kinds of stimuli and their meaning may not require as much effort to encode the information in the display as did our naive observers. Future work will be needed to establish whether overconfidence is observed in experienced operators.

Around 15% of detected changes were incorrectly identified (Table 1). This general result is consistent with signal detection theory where each symbol is represented by an independent change detector. On some change trials, it is possible that the (noisy) activation of the change detector representing the changed symbol may be lower than that of another detector representing an unchanged symbol. If the activation of any detector is above criterion, the change will apparently be detected even though the most highly activated detector represents the wrong symbol. Alternatively, it is possible that observers were sometimes aware of a change but unable to localise it. A recent study [33] has reported a similar difference between change detection and identification performance and interpreted this difference to reflect the detection of changes to the global statistics of the stimulus. A third possibility is that observers occasionally did not detect the changes, but instead made lucky guesses for detection and unlucky guesses for identification (note that the chance rate for detection is 1/2 while that for identification is 1/8).

## Experiment 2

The way that workers allocate their time and attentional resources varies between work environments. In many safety-critical domains, the evolution of the tactical environment places tight constraints on the allocation of time and attention. In such cases, workers must be cognisant of the available time and optimize their approach to the task accordingly (e.g., by favouring speed over accuracy for tight schedules). In other domains or during relatively quiet periods, time limits are self-imposed based on the worker’s assessment that the job has been completed to a sufficient or target standard.

Little is known about the alignment between objective and subjective assessments of performance in these different task contexts. Further, little is known about the effects of self-monitoring of knowledge for the purpose of regulating behaviour on task accuracy or metacognition. Experiment 2 addressed these questions by comparing objective and subjective performance across task contexts where study time was either controlled by the task (experimenter-paced) or was self-paced in order to satisfy specified performance targets. In the self-paced condition, observers are expected to have based their control of study time on a consideration of their judgements of learning (JOLs) and the nominated performance targets [4,34]. For both task contexts, we asked observers to report their JOLs after they had studied the initial display but before the change to the display. To increase the generalizability of the results to applied settings, Experiment 2 extended the range of display inspection times (1 to 15 seconds) and included potential changes to each of the symbol features (colour, shape, and leader line orientation).

### Method

#### Participants

Ten non-expert observers (eight men and two women) with normal or corrected-to-normal vision participated in this study. All were staff of the DSTO. All gave written informed consent before participating and were free to withdraw from the study at any time. The project was approved by the Chief of Air Operations Division, in accordance with the Australian National Statement on Ethical Conduct in Human Research [22].

#### Change localization task

The tactical displays were identical to those in Experiment 1, with the exception that only four symbols were presented. The mask interval was increased to 2 seconds and included a scale and mouse pointer on which observers indicted their JOLs as the perceived probability of correctly identifying an impending change on a continuous scale from .25 (chance) to 1.00 (certain). The comparison display included a change to the shape, colour or leader line orientation of one of the symbols and the observers' task was to indicate with the mouse which symbol had changed (a 4-alternative forced-choice task). Colour and shape changes involved changes from one member of the set to another. Line orientation changes involved rotations of the line by 90 degrees. The change localization response was not speeded. The display was then replaced by a uniform grey screen with a scale and mouse pointer on which observers indicated their JOP as the probability that they had correctly localised the change. No feedback was given. Displays were presented on a 19” CRT monitor (Barco CCID121) with a frame rate of 60 Hz.

#### Design

There were two task contexts (experimenter paced, self paced). In the experimenter-paced condition, the inspection time of the initial display was terminated automatically by the software after 1, 2, 3, 6, 9, 12, or 15 seconds, in separate blocks. In the self-paced condition, observers were instructed to terminate the initial display after studying it for only as long as they felt was required to achieve a performance target nominated by the experimenter (0.4, 0.5, 0.6, 0.7, 0.8, or 0.9 correct, in separate blocks). For all analyses, these performance targets were taken to be the JOLs for the self-paced condition. However, to ensure that the task demands of the self-paced condition were maximally similar to the experimenter-paced condition, observers also indicated these JOLs (i.e., target performance) during the mask period.

Each block contained 60 trials and an equal number of colour, shape, and line orientation changes, presented in random order. The order of completion of blocks was counterbalanced across observers. The order of completion of conditions of control (self paced, experimenter paced) was counterbalanced across observers.

Data were compared across conditions of judgement (JOL, JOP) and control (self paced, experimenter paced). Data were pooled across conditions of change type for three reasons. First, JOLs were made before a change had occurred and therefore could not have incorporated any knowledge about the type of change in a trial. A rational observer would therefore base their JOLs on their perceived performance averaged across change types. Second, separate calibration curves were generated for each observer and there were not sufficient data to generate reliable calibration curves for each condition of change type, judgement, and control. Third, any comparisons across change types would be limited to the specific stimuli employed. That is, the salience of each change type would not be expected to generalise to other changes of the same type.

The results of experiment 2 (a 4-alternative forced-choice task) could not be analysed using meta *d'* because the method is currently applicable only to 2-alternate forced-choice detections [32]. JOLs and JOPs were sorted into 6 bins of width .125 from .25 (chance) to 1.0 (perfect performance). As stated above, the performance targets (.4, .5, .6, .7, .8, .9) were taken to be the JOLs for the self-paced condition.

#### Statistical analysis

Means were analysed using paired *t*-tests or repeated-measures analyses of variance (ANOVAs). An alpha level of .05 was used for all statistical tests.

### Results

#### Objective change localisation performance

**Fig 4:**
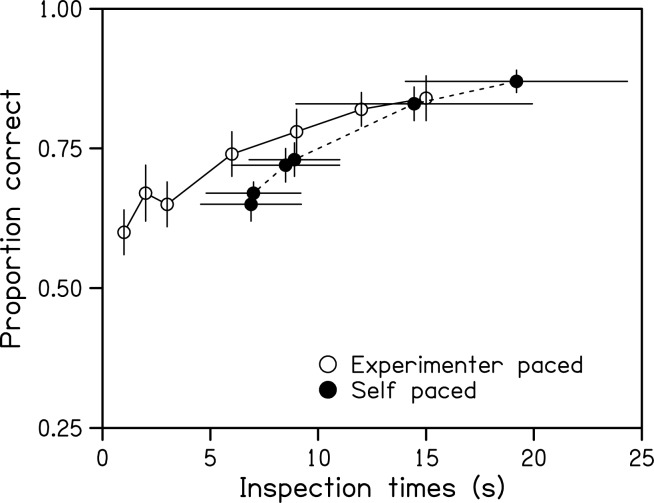
Change localisation performance. Change localization performance as a function of inspection time for the experimenter-paced and self-paced conditions in Experiment 2. Errors bars indicate standard errors of the means.

Mean objective performance, collapsed across change types, is shown for the experimenter-paced and self-paced conditions in Fig 4. As expected, observers modulated their performance in the self-paced condition by adjusting their inspection times. Objective performance in both conditions increased with inspection time and converged for the longer inspections and higher performance levels. For shorter inspections and lower performance levels, the curve for the self-paced condition was shifted to the right by about three seconds compared to that for the experimenter-paced condition. Average accuracy, pooled across inspection times (Table 3), was not significantly different between conditions of control, *t*(9) = 0.56, *p* = .59. Similarly, average type-1 *d'* was not significantly different between conditions of control, *t*(9) = 0.06, *p* = .95.

**Table 3:** Objective accuracy and metrics of metacognition in change detection for Experiment 2.

	Judgement	Experimenter-paced	Self-paced
**Accuracy**	*-*	.73 (.04)	.75 (.02)
**Type-1 *d'***	*-*	1.67 (0.17)	1.68 (0.07)
**Av. confidence error**	JOL	-.117 (.016)	-.096 (.018)
	JOP	-.036 (.014)	-.032 (.019)
**Calibration**	JOL	.023 (.005)	.025 (.004)
	JOP	.006 (.001)	.007 (.001)
**Normalised resolution**	JOL	.065 (.016)	.054 (.009)
	JOP	.268 (.034)	.284 (.024)

Note: Standard errors of the means are shown in parentheses.

#### Metacognitive monitoring and control

**Fig 5:**
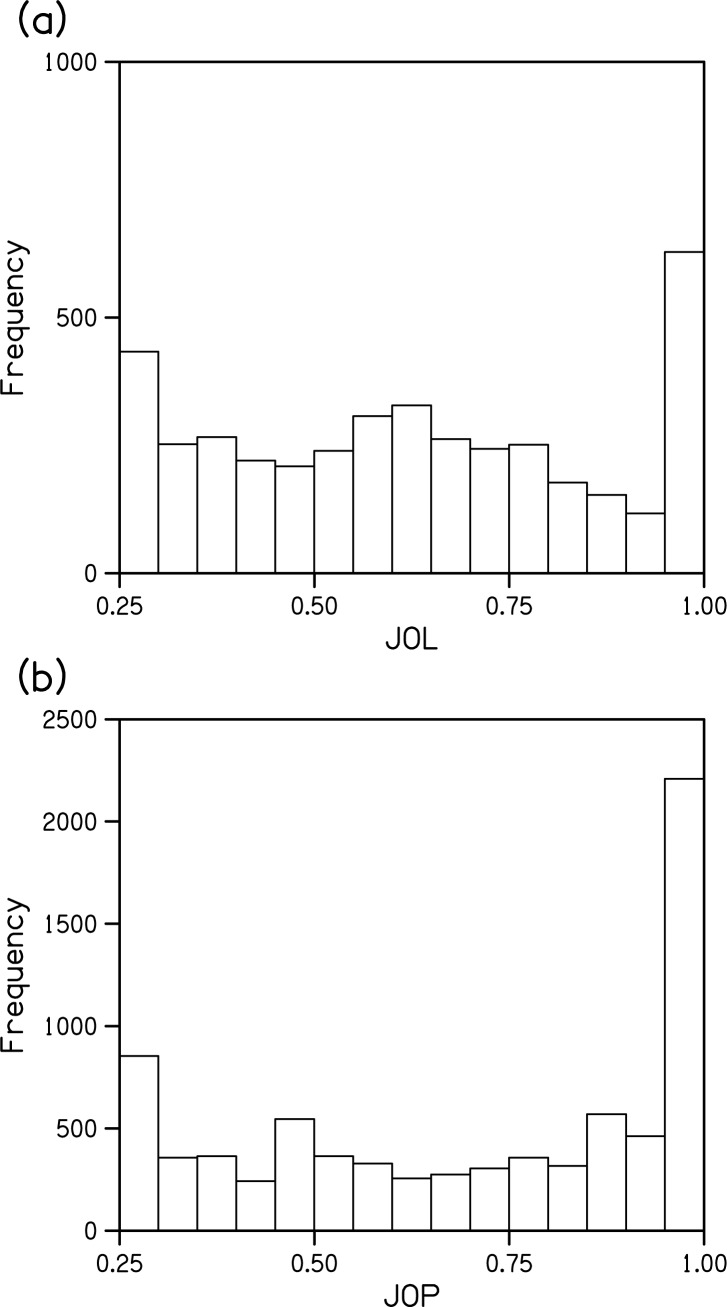
Distribution of JOLs and JOPs. (a) Histogram of JOLs in the experimenter-paced condition. (b) Histogram of JOPs pooled across self-paced and experimenter-paced conditions.

Examination of the distributions of JOLs and JOPs indicated that observers experienced a continuum of certainty in this task (Fig 5). However, extreme levels of certainty and uncertainty were common with 21% of JOLs and 32% of JOPs being equal to 0.25 (chance) or 1.0 (certain). Change localization was correct on 96% of JOLs and 98% of JOPs that were judged to be certain. Localization was correct on 54% of JOLs and 35% of JOPs that were judged to be at chance (25%).

**Fig 6:**
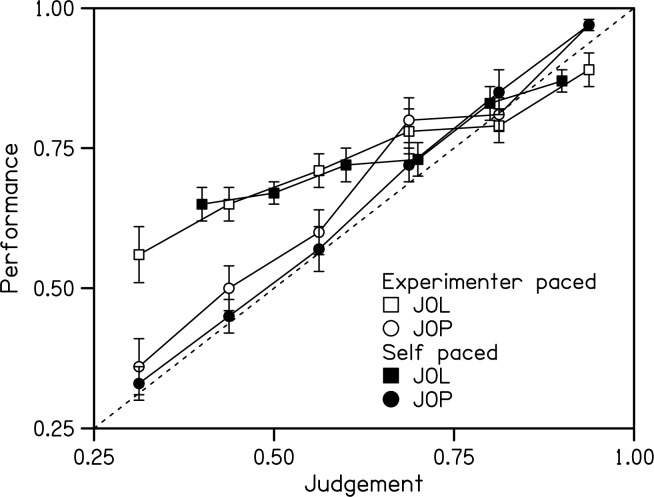
Calibration curves. Calibration curves for JOLs and JOPs in the self-paced and experimenter-paced conditions of control in Experiment 2. Errors bars indicate standard errors of the means.

Inspection of Fig 6 indicates that calibration curves for JOLs and JOPs were similar for both self-paced and experimenter-paced conditions. JOLs less than 0.7 were underconfident (above the diagonal), but JOPs were well calibrated across the range of judgements.

The average confidence error of JOLs in both conditions of control and of JOPs in the experimenter-paced condition were significantly different from zero in the direction of underconfidence, *t*(9) ≥ 2.49, *p* ≤ .034. Average confidence error was analysed with a 2-way repeated-measures ANOVA with variables of Control (experimenter paced, self paced) and Judgement (JOL, JOP). The main effect of Judgement was significant, *F*(1,9) = 38.9, *p* < .001, *η*_*p*_^*2*^ = .80, with JOPs significantly less underconfident than JOLs. The main effect of Control was not significant, *F*(1,9) = 0.71, *p* = .42, *η*_*p*_^*2*^ = .07, and the interaction between these variables was not significant, *F*(1,9) = 1.28, *p* = .29, *η*_*p*_^*2*^ = .12.

Average calibration was analysed with a 2-way repeated-measures ANOVA with variables of Control (experimenter paced, self paced) and Judgement (JOL, JOP). The main effect of Judgement was significant, *F*(1,9) = 38.8, *p* < .001, *η*_*p*_^*2*^ = .81, with better calibration for JOPs than for JOLs. The main effect of Control was not significant, *F*(1,9) = 0.35, *p* = .57, *η*_*p*_^*2*^ = .04, and the interaction between these variables was not significant, *F*(1,9) = 0.14, *p* = .72, *η*_*p*_^*2*^ = .01.

Average normalised resolution was analysed with a 2-way repeated-measures ANOVA with variables of Control (experimenter paced, self paced) and Judgement (JOL, JOP). The main effect of Judgement was significant, *F*(1,9) = 69.7, *p* < .001, *η*_*p*_^*2*^ = .89, with better resolution for JOPs than for JOLs. The main effect of Control was not significant, *F*(1,9) = 0.006, *p* = .94, *η*_*p*_^*2*^ = .001, and the interaction between these variables was not significant, *F*(1,9) = 0.38, *p* = .55, *η*_*p*_^*2*^ = .04.

### Discussion

#### Objective performance

The displays in Experiment 2 comprised only four symbols. Given the sparseness of these displays, observers demonstrated a surprisingly low level of SA, even when they were allowed long periods for study. For example, in the self-paced condition, inspection times of around seven seconds resulted in an average change localization rate of only 65% (Fig 3). Accuracy only increased to around 90% when displays were studied for approximately 20 seconds. These results are in contrast to those of another study [14] which reported a detection accuracy of around 90% for very short (0.1-second) exposures to displays of four symbols where a subsequent change could occur to any of four features (gap, size, orientation, colour) of any of the symbols. Luck and Vogel interpreted their result to indicate that VSTM encodes around four integrated objects rather than conjunctions of individual features. It is possible that observers in our study did not similarly integrate symbol features into integrated objects.

While change localisation performance reflects limitations in VSTM [13], the operational impact of VSTM limitations may be reduced if operators are able to rely on the enduring nature of visual information presented on their displays to serve as an unlimited capacity "external memory" which may be accessed as necessary for a task [35]. Failures to retrieve the required information from VSTM are expected to prompt further inspection of the displays. This requires the location where information may be found to be held in memory, but for many tasks this is more enduring than the information itself. Limitations of VSTM will, however, limit observers' timely awareness of changes to their displays unless they are accompanied by visible transients which attract attention.

#### Subjective judgements

In the condition where inspection time was experimenter paced, it is not clear whether observers continuously monitored their JOLs during the inspection of the initial display or whether they waited until it terminated before assessing their JOLs. In the self-paced condition, observers were required to continuously monitor their JOLs in order to make decisions to terminate their inspections of the display.

Objective change localisation performance did not differ between experimenter-paced and self-paced conditions, suggesting the visual information was similarly learned. However for performance targets of 0.7 or less in the self-paced condition, observers required longer inspections to reach similar levels of objective performance. It is therefore possible that the requirement in the self-paced condition to simultaneously learn the display and monitor JOLs resulted in either a slowing of encoding or an interference with the maintenance of symbols or features in VSTM. This result is in contrast to that of a recent study [36] where word pairs were *better* recalled when a JOL was made, suggesting the act of making the JOL increased the salience of the cues underling memory performance. That the average confidence error, calibration and resolution of JOLs did not differ between self-paced and experimenter-paced conditions suggests that observers had similar levels of insight in each of these conditions about the degree to which the visual information had been learned.

JOPs were less underconfident, better calibrated and better resolved than JOLs. As JOLs were elicited before the change, they were made under conditions of uncertainty about the type of change that would occur. JOPs were made following the change and therefore in the presence of information with regard to the salience of the actual change that occurred. Also, while JOPs could be made with reference only to performance on the current trial, JOLs are likely to reflect an integration of the perceived current state of knowledge with JOLs and JOPs across previous trials.

## General discussion

This study examined metacognitive monitoring and control in change detection tasks using displays that mimic those used in a range of operational roles, such as air-traffic control and military command and control. Although the stimuli were comparable to applied work environments, the scope of this study was restricted to non-expert observers to examine fundamental aspects of metacognition in such tasks. Thus, this study provides comparative data and a methodological basis to extend this work to expert populations in future studies.

Experiments 1 and 2 showed that observers had limited SA of the tactical information presented on their displays. In Experiment 2, the tactical situation represented in the displays was particularly sparse, with only four entities shown. Nevertheless, accuracy was only 65% with inspections of around seven seconds—a long time in the context of many operational tasks. This poor performance suggests that observers in our study did not efficiently bind symbol features into integrated objects. It is possible that experienced operators who are familiar with these stimuli and their semantic content may perform better in the change detection task (reflecting a higher SA) due to an ability to interpret the stimuli in the context of existing knowledge schemas, e.g., “a friendly pair travelling north” instead of “two blue open rectangles with lines oriented vertically”.

While observers' knowledge of the tactical situation was limited, they demonstrated a good understanding of the limitations of that knowledge; at least in terms of JOPs. In Experiment 1, meta *d'* was close to type-1 *d'*, consistent with the interpretation that observers' metacognition with regard to their detection performance was close to optimal, given the limitations imposed by their type-1 sensitivity. Also, in Experiment 2 JOPs and JOLs for high performance targets were well calibrated. In contrast to previous studies of change detection, there was no evidence of “change blindness blindness” in the current study. Instead, subjective assessments were either approximately accurate or *under*-confident. This difference could potentially be reconciled with reference to the effort heuristic, which posits that items that require more effort to study are judged to be less well learned. Specifically, it is possible that while both novices and experts have difficulty predicting their own performance on tasks of this kind, the development of context-specific schemas and the associated changes in perceived ease of learning moderate the size and direction of this effect; engendering a tendency towards *under*-confidence in those with little experience and towards *over*-confidence in those with a great deal of experience. Furthermore, experts' beliefs about their ability to extract meaning from their displays may also modulate confidence [20]. These possibilities are consistent with the fact that most studies demonstrating *over*-confidence in change detection ability have been conducted using stimuli that represent natural scenes. Future work will be needed to establish whether overconfidence is observed in experienced operators. Nevertheless, our results demonstrate that change blindness blindness is not ubiquitous in change detection tasks and suggests that the symbolic displays used in these operational environments might be less susceptible to the phenomenon than the perception of natural scenes.

The absence of overconfidence is also in contrast to the overconfidence typically observed for general knowledge (see [37] for a review). While some studies, e.g., [26,38], have reported similar calibrations of confidence for perception and knowledge, others (e.g., [39]) have reported that judgements for sensory decisions are consistently underconfident, and suggested that the process underlying confidence judgements for perceptual decisions may be different from that underlying confidence judgements for decisions about knowledge. The present study examined confidence in short-term memory, which is intermediate between perception and long-term memory (knowledge), and found JOLs and JOPs to be either approximately accurate or underconfident.

In Experiment 2, observers were assigned different performance targets for accuracy across trial blocks and used their JOLs to control their study time. That observers regulated their study time to meet performance targets is consistent with agenda-based regulation where agendas for study (i.e., the allocation of cognitive resources during study) are modulated in response to task goals [34]. However on average, this requirement for self-monitoring and control of study was associated with an efficiency cost: observers studied the displays for longer to achieve comparable levels of performance to those in the experimenter-paced condition. This effect, however, was more evident for the blocks in which the performance targets were low (and inspection times short) and diminished for the high performance targets (where inspection times were long).

These results suggest two important conclusions in the context of work environments. First, operators working under self-paced conditions must be cognisant of the diminishing value in further study beyond a certain point (i.e., the 'labor-in-vein effect'; [40]). Given that observers in our task demonstrated good metacognition in the asymptotic part of the time-performance curve, it may be possible to encourage them to recognise this point as a cue to progress to the next task. Second, where possible, the requirements to monitor knowledge and to regulate study could be eliminated, particularly for those task components that can be achieved in a manner of a few seconds where the cost of self-monitoring is greatest.

In many work environments, timely feedback is often not available. For example, an air traffic controller may not be immediately warned that he/she had missed a new contact. Operators must instead rely on their metacognitive assessments of their knowledge and performance. Where metacognitive judgements are found to be poorly calibrated, training paradigms may be devised to provide feedback in order to improve calibration and resolution [41], and therefore to also improve objective performance through the more appropriate control of attentional allocation. It may also be possible to improve metacognitive control by explicit training to raise awareness of errors of metacognition (cognitive debiasing), including heuristics and biases [42], as has been advocated for reducing diagnostic errors by doctors in emergency departments [43], or by providing strategies for critiquing and correcting gaps in one’s own understanding (e.g., [2]).

### Limitations of the study

As stated above, the present study employed observers who were naïve with respect to the semantic content of the symbols. Further research is required to investigate how the semantic knowledge of experts modulates change detection and metacognition.

The generation of robust calibration curves for each observer requires the collection of several data points for each confidence bin. Each observer completed 480 or 780 trials for Experiments 1 and 2, respectively. Each experiment recruited 10 observers. While samples of this size are not uncommon in repeated-measures designs in psychophysics, we acknowledge that larger samples would provide greater power to detect small effects. We note that the vast majority of the non-significant effects reported here were of small or negligible size. The study had sufficient power to detect the highly significant effects reported. Considering the significant ANOVA results, all but one are reliable with a probability of replication (*p*_*rep*_) of .99 or greater. The remaining result (the effect of Change on average confidence error in Experiment 1) has a *p*_*rep*_ of .93.
